# Equity and Coverage in the Continuum of Reproductive, Maternal, Newborn and Child Health Services in Nepal-Projecting the Estimates on Death Averted Using the LiST Tool

**DOI:** 10.1007/s10995-019-02828-y

**Published:** 2019-11-30

**Authors:** Jeevan Thapa, Shyam Sundar Budhathoki, Rejina Gurung, Prajwal Paudel, Bijay Jha, Anup Ghimire, Johan Wrammert, Ashish KC

**Affiliations:** 1 School of Public Health and Community Medicine, B.P Koirala Institute of Health Sciences, Dharan, Nepal; 2 Golden Community, Lalitpur, Nepal; 3 Ministry of Health and Population, Government of Nepal, Kathmandu, Nepal; 4 Nepal Health Research Council, Kathmandu, Nepal; 5 Department of Women’s and Children’s Health, Uppsala University, Uppsala, Sweden; 6 International Maternal and Child Health, Department of Women’s and Children’s Health, University Hospital, 751 85 Uppsala, Sweden

**Keywords:** Countdown to 2030, Death averted, Maternal, neonatal and child survival, Nepal, Sustainable development goal

## Abstract

**Introduction:**

The third Sustainable Development Goal, focused on health, includes two targets related to the reduction in maternal, newborn and under-five childhood mortality. We found it imperative to examine the equity and coverage of reproductive, maternal, newborn and child health (RMNCH) interventions from 2001 to 2016 in Nepal; and the death aversion that will take place during the SDG period.

**Methods:**

We used the datasets from the Nepal Demographic Health Surveys (NDHS) 2001, 2006, 2011 and 2016. We calculated the coverage and equity for RMNCH interventions and the composite coverage index (CCI). Based on the Annualized Rate of Change (ARC) in the coverage for selected RMNCH indicators, we projected the trend for the RMNCH interventions by 2030. We used the Lives Saved Tools (LiST) tool to estimate the maternal, newborn, under-five childhood deaths and stillbirths averted. We categorised the interventions into four different patterns based on coverage and inequity gap.

**Results:**

Between 2001 and 2016, a significant improvement is seen in the overall RMNCH intervention coverage-CCI increasing from 46 to 75%. The ARC was highest for skilled attendance at birth (11.7%) followed by care seeking for pneumonia (8.2%) between the same period. In 2016, the highest inequity existed for utilization of the skilled birth attendance services (51%), followed by antenatal care (18%). The inequity gap for basic immunization services reduced significantly from 27.4% in 2001 to 5% in 2016. If the current ARC continues, then an additional 3783 maternal deaths, 36,443 neonatal deaths, 66,883 under-five childhood deaths and 24,024 stillbirths is expected to be averted by the year 2030.

**Conclusion:**

Nepal has experienced an improvement in the coverage and equity in RMNCH interventions. Reducing inequities will improve coverage for skilled birth attendants and antenatal care. The current annual rate of change in RMNCH coverage will further reduce the maternal, neonatal, under-five childhood deaths and stillbirths.

**Electronic supplementary material:**

The online version of this article (10.1007/s10995-019-02828-y) contains supplementary material, which is available to authorized users.

## Significance

Improving equity and coverage of reproductive, maternal, neonatal and child survival interventions will be critical to reduce preventable deaths globally and in Nepal. We used Nepal’s demographic and health survey data of 2001, 2006, 2011 and 2016 to provide the trend in the coverage estimate of RMNCH interventions and equity profile of the coverage. The key to avert the preventable deaths lies upon the reduction of the inequity gap and sustaining the improvement in the services coverage.

## Introduction

The Sustainable Development Goal for health (SDG 3) has set several targets in its bid to accelerate and sustain the momentum for reducing preventable maternal, neonatal and child deaths (Sachs 2012; United Nations General Assembly 2015). Globally, maternal deaths havedecreased slightly from 390,185 in 1990 to 275,288 in 2015 (Kassebaum et al. 2016). The annual rate of reduction has been 1.5% indicating a state of sluggishness or stagnation of maternal deaths between 1990 and 2015 (You et al. 2015). With a reduction rate of 2.4% in the same period, approximately 92,000 mothers die each year in South Asia (Kassebaum et al. 2016). In Nepal, Maternal Mortality Ratio (MMR) has decreased from 529 to 239/100,000 live births between 1990 and 2015 (Kassebaum et al. 2016; Ministry of Health, Nepal 2017).

Around 5.8 million deaths among children below 5 years old were observed globally in 2015, accounting for a 52.0% decrease in the under-five childhood mortality since 1990 (Wang et al. 2016). In South Asia, an estimated 50 under-five childhood deaths per 1000 live birth, 21 stillbirths per 1000 birth and 30 neonatal deaths per 1000 live birth were reported in 2015. Between 1990 and 2015, the under-five childhood mortality decreased by 3.42% each year. In Nepal, the Annual Rate of Reduction (ARR) for the under-five childhood mortality was 5.22% between the same time period (Wang et al. 2016).

The reproductive, maternal, neonatal and under-five childhood mortality and stillbirths can be prevented through various interventions (Bhutta et al. 2014; Vaivada et al. 2017). These interventions range from family planning to prevent unwanted pregnancy, antenatal care, vaccination against tetanus toxoid, skilled attendance at birth, BCG vaccination, DPT3 vaccination, measles vaccination, treatment of diarrhea, care seeking for pneumonia, vitamin-A supplementation and access to improved water sources (Barros et al. 2012; Boerma et al. 2008). The countdown for 2015 and 2030 reports on the coverage of these interventions from the preconception period till the fifth year of the child’s life (Coverage and Group 2015; Horton 2008).

This study assessed the equity and coverage of reproductive, maternal, newborn and child health (RMNCH) interventions from 2001 to 2016 in Nepal. Based on the trend in the coverage of RMNCH interventions, the study also aimed to estimate the maternal deaths, neonatal deaths, under-five childhood deaths and stillbirths that could be averted during the SDG period from 2016 to 2030.

## Methods

This is a secondary analysis of the quantitative data from the demographic health surverys. We utilised the datasets from the Nepal Demographic Health Surveys of 2001, 2006, 2011 and 2016 (Ministry of Health and Population, Nepal 2002; Ministry of Health, Nepal 2007; Ministry of Health and Population, Nepal 2012; Ministry of Health, Nepal 2017).

These datasets contain a nationally representative data of ever-married women of reproductive age group of 15–49 years and men of 15–59 years obtained through surveys at national, regional and sub-regional levels, in urban and rural areas respectively.

In 2001 NDHS, the 1991 census was used as the population frame with 13 sampled domains and 8400 ever-married women of 15–49 years of age and 8700 households were selected nationwide. In 2006 NDHS, the 2001 census was used as the population frame with 13 sampled domains and 8600 ever-married women of 15–49 years of age and 9036 households were selected nationwide. In 2011 NDHS 2001 census was used as the population frame with 13 sampled domains and 13,485 ever-married women of 15–49 years of age and 11,085 households were selected nationwide. In 2016 NDHS, the 2011 census was used as the population frame, and the country was divided into 14 domains, 383 clusters, 4235 eligible men, 13,089 ever-married women age 15–49 and 11,473 households were selected nationwide.

These datasets have information on the socio-economic status, place of residence, ecological location, education, maternal age, wealth quintile, service accessed and utilized by women during pre-pregnancy, pregnancy, delivery, postnatal and child care.

### Data Management

The following variables were extracted from all four datasets for this study- Contraceptive prevalence rate using modern method (FP), Unmet need for family planning, Family planning need satisfied, One antenatal check-up from skilled providers (ANC), Vaccination against tetanus (Td), Skilled attendance at birth (SBA), BCG vaccination, Diphtheria Pertussis and tetanus (DPT) three dose vaccination (DPT3), Measles rubella vaccination (MSL), Case management of diarrhoea (ORT), Care seeking for pneumonia (CPNM), Vitamin A supplementation and Access to clean water.

### Data Analysis

We used SPSS 23.0 version for the analysis of the coverage and equity calculation. We generated the coverage estimate with reference from the Countdown to 2030 reporting of RMNCH indicators (Boerma et al. 2018). We calculated the composite coverage index with the reference from the Countdown 2015 report (Boerma et al. 2008).

The Composite coverage index (CCI) uses a weighted mean of the coverage of eight interventions selected from four specialties (family planning, maternity care, child immunization, and case management) (Boerma et al. 2008).

$$CCI = \frac{1}{4}\left( {FP + \frac{SBA + ANC}{2} + \frac{2DPT3 + MSL + BCG}{4} + \frac{ORT + CPNM}{2}} \right)$$

We presented a trend of the RMNCH interventions coverage for years 2001, 2006, 2011 and 2016. The DHS survey uses the first principal component analysis to generate the wealth quintile based on the house asset possession score. Data on asset ownership (e.g. owning a bicycle or radio) and housing characteristics (e.g. number of rooms or type of toilet facilities), which are called “asset indicators” were used to construct an “asset index” and to establish cut-off values for wealth quintiles within the population. These quintiles were then ranked from bottom to top as poorest, poorer, middle, wealthier and wealthiest (Filmer and Pritchett 2001). We used the coverage of the RMNCH interventions among the poorest and wealthiest quintile to generate the equi-plot for 2001 and 2016. For analysis of inequity, we calculated the ratio and difference between the poorest and wealthiest population strata and the coverage estimate for RMNCH interventions by maternal education (no education or at least primary education) and place of residence (urban or rural) for 2016. Based on coverage and inequity gap, we categorised the interventions into four different patterns, namely, high coverage low inequity, high coverage high inequity, low coverage low inequity and low coverage high inequity.

For Nepal, we estimated the RMNCH interventions coverage using the datasets of the NDHS 2016. We calculated the annualized rate of change (ARC) in the coverage of the RMNCH interventions based on the coverage trend from 2001 till 2016.

$${\text{ARC}} = - \left( { \left( {\frac{{{\text{Intervention}}\;t2}}{{{\text{Intervention}}\;t1}}} \right)^{{\frac{1}{t2 - t1}}} - 1} \right) \times 100$$

where t_2_ is the end year and t_1_ is the beginning year. An ARC from 2001-2016 would therefore be calculated as—$\left( {\left( {\left( {{{{\text{Intervention}}_{2016} } \mathord{\left/ {\vphantom {{{\text{Intervention}}_{2016} } {{\text{Intervention}}_{2001} }}} \right. \kern-0pt} {{\text{Intervention}}_{2001} }}} \right)^{(1 /15)} } \right) - 1} \right) \times 100$. Based on the ARC between 2001 and 2016, we projected coverage for Nepal by 2030.

We used the Lives Saved Tool (LiST) to estimate the maternal, neonatal, under-five childhood deaths and stillbirths averted using the coverage estimate of the interventions (Boschi-Pinto and Black 2011; Walker et al. 2013). LiST is a demographic software package projects population trends using the trends of last 20 years based on UN estimates (Boschi-Pinto et al. 2010). LiST includes modules for the impact of various factors on demography, including a module for maternal, and child health. The LiST has previously been used to measure the impact of interventions on maternal and child health (Winfrey et al. 2011).

The model works by describing a country or region in terms of its demography, health status such as stunting rates, exposure to falciparum malaria, rates and causes of mortality and the current coverage of interventions for a baseline year, normally the current year (Fox et al. 2011). The software then combines the baseline information along with the scale up and the effectiveness of the interventions to estimate the impact of these different scenarios on maternal, neonatal and child health (Walker and Friberg 2017). The estimates of the lives saved are modelled in such a way that the lives cannot be saved twice by the linked interventions. The calculation of impact of interventions is based on the time within the continuum of care where by changes in the coverage reduces the number of cases and deaths (Boschi-Pinto and Black 2011; Walker et al. 2013).

## Results

Between 2001 and 2016, an increase in Composite Coverage Index (CCI) from 46 to 75% in the overall RMNCH intervention coverage is seen. The improvement in coverage is more than threefold for the interventions, such as antenatal care, skilled attendance at birth and care seeking for pneumonia. The intervention coverage for use of modern family planning method and treatment for diarrhoea has not improved significantly despite the coverage being low in 2001. The ARC is highest with skilled attendance at birth (11.7%) followed by care seeking for pneumonia (8.2%). The slowest rate of change was seen with access to improved water sources (0.7%) followed by family planning need satisfied (0.9%) (Table 1).

**Table 1:** Coverage of reproductive, maternal, newborn and child health interventions in 2001, 2006, 2011 and 2016

Interventions	2001 (%)	2006 (%)	2011 (%)	2016 (%)	2016–2001 (%)	ARC (%)
Composite Coverage Index	46	56	63	75	29.1	3.3
CPR of modern method	35	44	43	43	7.4	1.3
Unmet need	28	25	28	24	− 4.1	− 1.1
Family planning need satisfied	56	64	61	64	8.3	0.9
SBA assisted delivery	12	20	41	63	50.8	11.7
ANC by skilled provider	28	44	59	86	57.4	7.7
DPT-HiB-Hb vaccination	72	89	92	86	13.8	1.2
Measles rubella vaccination	71	85	88	90	19.8	1.7
Tetanus toxoid 2+ doses	46	64	69	65	19.6	2.4
BCG vaccination	85	93	97	98	13.0	1.0
All basic vaccination	66	83	87	78	12.2	1.1
Treatment of diarrhea by ORT and continued feeding	42.6	36.8	46.7	61.4	18.8	2.5
Care seeking for pneumonia	26.1	42.9	49.5	84.9	58.8	8.2
Vitamin A supplementation	–	88	87	83		
Access to improved water source	85	85	92	95	9.2	0.7

Basic vaccination-BCG, 3 doses of oral polio, 3 doses of DPT, 1 dose of measles-rubella. All basic vaccination includes all those in the expanded programme for immunization in Nepal

 In 2016, the inequity gap for utilization of skilled attendance at birth was observed by the place of residence (18.6%) and educational status (30%). Inequity was observed in utilization of all basic vaccination services (14.1%) and treatment of diarrhoea with ORT and continuous feeding (15.3%) in 2016 (Tables 2, 3).

**Table 2:** RMNCH coverage by place of residence in 2016

Interventions	Place of residence		Urban–rural difference	
	Urban	Rural	Difference	Ratio
Composite Coverage Index	77.9	72.5	5.4	1.07
CPR of modern method	44.2	40.6	3.6	1.1
Unmet need	22.7	25.3	− 2.6	0.9
Family planning need satisfied	66.1	61.6	4.5	1.1
ANC by skilled provider	87.7	83.0	4.7	1.1
Tetanus toxoid 2+ doses	67.3	63.2	4.1	1.1
SBA assisted delivery	71.4	52.8	18.6	1.4
BCG vaccination	98.1	96.8	1.3	1.0
DPT-HiB-Hb vaccination	85.6	86.3	− 0.7	1.0
Measles rubella vaccination	91.2	89.5	1.7	1.0
All basic vaccination	78.5	77.0	1.5	1.0
Vitamin A supplementation	82.1	83.0	− 0.9	1.0
Treatment of diarrhea by ORT and continued feeding	61.8	60.8	1.0	1.0
Care seeking for pneumonia	89.7	80.7	9.0	1.1
Access to improved water source	93.7	96.1	− 2.4	1.0

Basic vaccination-BCG, 3 doses of oral polio, 3 doses of DPT, 1 dose of measles-rubella. All basic vaccination includes all those in the Expanded programme for immunization in Nepal

**Table 3:** RMNCH coverage by maternal education in 2016

Interventions	Maternal education		Educated-uneducated difference and ratio	
	Yes	No	Difference	Ratio
Composite Coverage Index	77.2	73.2	4	1.1
CPR of modern method	34.1	48.8	− 14.7	0.7
Unmet need	28.2	20.6	7.6	1.4
Family planning need satisfied	54.7	70.3	− 15.6	0.8
ANC by skilled provider	90.2	80.8	9.4	1.1
Tetanus toxoid 2+ doses	73	57.8	15.2	1.3
SBA assisted delivery	78	47.8	30.2	1.6
BCG vaccination	98.6	96.4	2.2	1.0
DPT-HiB-Hb vaccination	90	82.2	7.8	1.1
Measles rubella vaccination	94.3	86.8	7.5	1.1
All basic vaccination	85.1	71	14.1	1.2
Vitamin A supplementation	84.2	81.2	3	1.0
Treatment of diarrhea by ORT and continued feeding	70.6	55.3	15.3	1.3
Care seeking for pneumonia	82.5	87	− 4.5	0.9

Basic vaccination-BCG, 3 doses of oral polio, 3 doses of DPT, 1 dose of measles-rubella. All basic vaccination includes all those in the expanded programme for immunization in Nepal

Based on the coverage and inequity in RMNCH interventions, we observed the following patterns:

### High coverage and reduced inequity

**Fig. 1:**
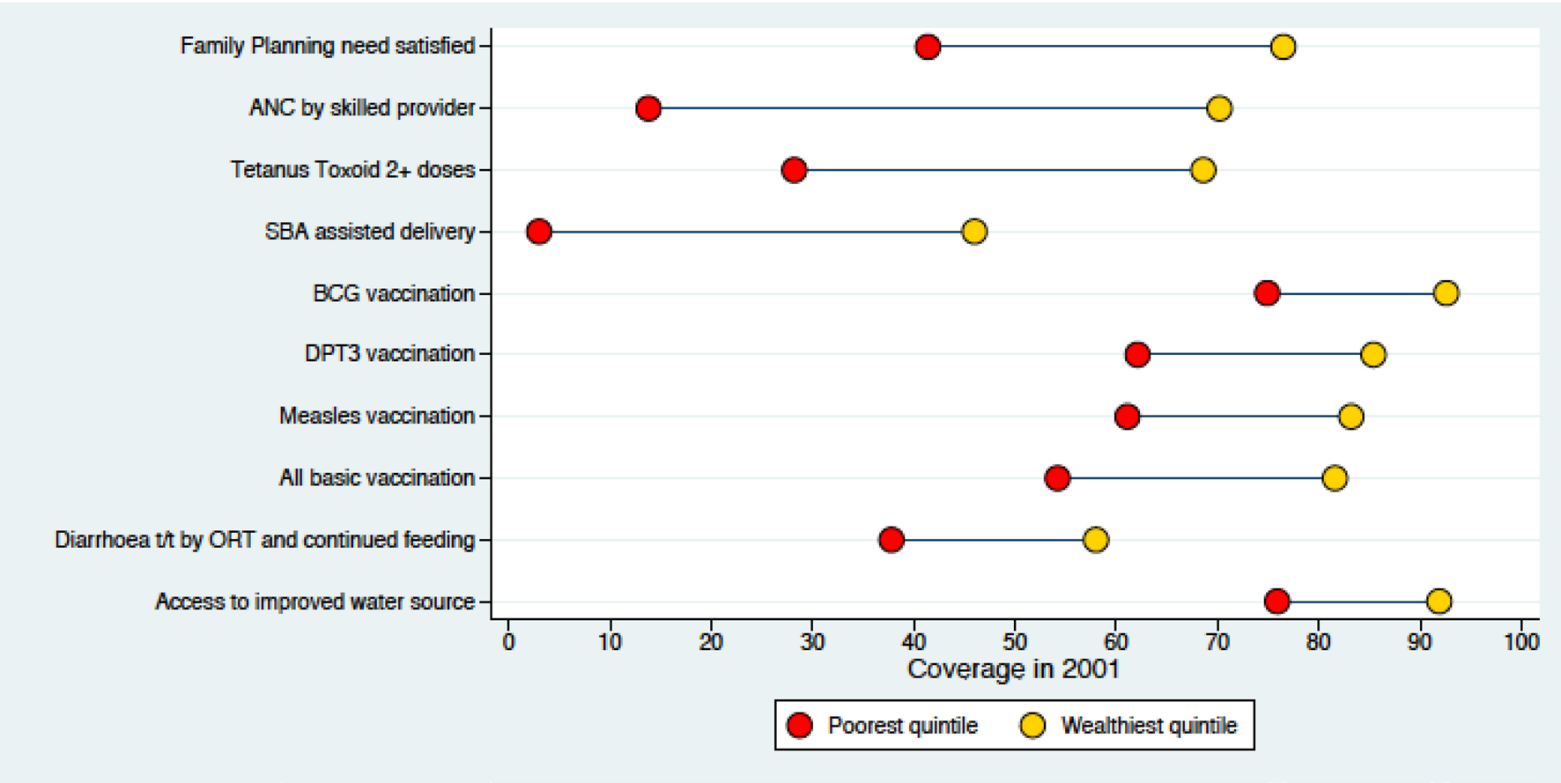
Coverage of RMNCH in different wealth quintile in 2001

**Fig. 2:**
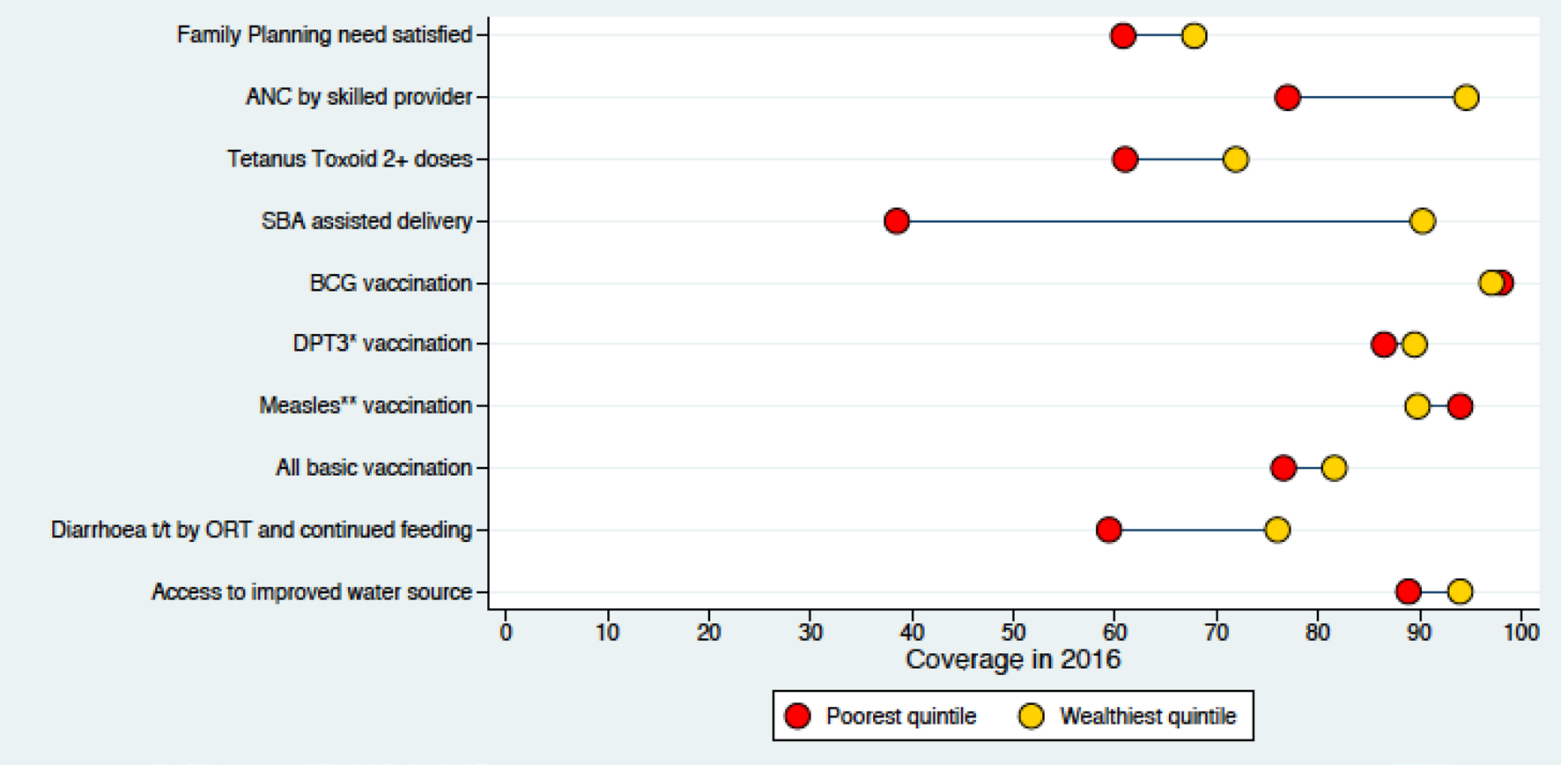
Coverage of RMNCH in different wealth quintile in 2016

 In 2016, the immunization coverage was high with measles-rubella vaccination at 90% and inequity gap of − 4.2% (Figs. 1, 2).

### High Coverage and High Inequity

In 2016, the coverage for ANC by skilled provider was 86% with inequity gap of 17.6% (Figs. 1, 2).

### Low Coverage and Reduced Inequity

In 2016, the coverage for family planning was 64% with inequity gap of 6.9% (Figs. 1, 2).

### Low Coverage and High Inequity

In 2016, the coverage for treatment of diarrhoea by ORT was 61.4% with inequity gap of 16.6% (Figs. 1, 2).

**Fig. 3:**
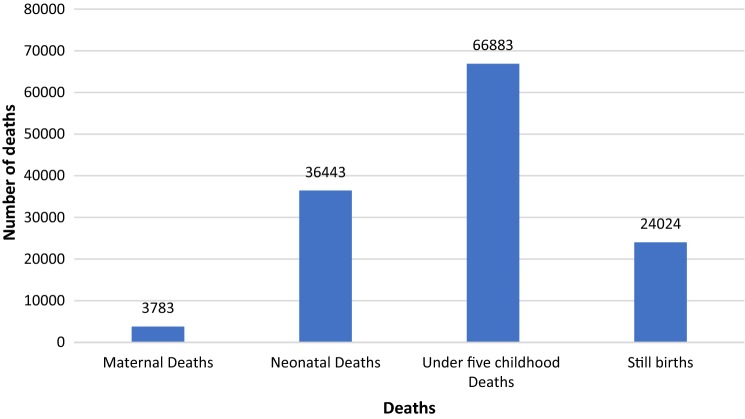
Maternal, neonatal, under-five deaths and stillbirth averted by 2030

Our estimate based on the LiST tool is that if the current ARC continues, an additional 3783 maternal deaths, 36,443 neonatal deaths, 66,883 under-five childhood deaths and 24,024 stillbirths will be averted (Fig. 3). The stillbirth rate will decrease from the current 18 to 13 per 1000 birth, neonatal mortality rate from 21 to 12 per 1000 live birth, post-neonatal from 17 to 7 per 1000 live birth by 2030.

## Discussion

 The coverage of all reproductive, maternal, newborn and child health services improved over the period of 15 years (2001–2016). For immunization services, while the coverage has not improved significantly in last 15 years, a significant reduction is seen in the inequity gap. At the start of the twenty first century, more than 90% of the babies in the highest wealth quintile received vaccination services and a wide disparity among children was noted in the lower quintiles. At the end of MDG era, the vaccination coverage for immunization services has improved significantly even among the children from the poorest quintiles. Eventually, the inequity gap is decreased as a result of accelerated annualized change in the coverage among the poor and the slow rate of change among the wealthy ones. For maternal health services, we noticed a four fold increase in the use of skilled birth attendance in last 15 years. The equity gap in 2001 was low because the maternal health services coverage was low for both poorest and highest wealth group. However, in 2016, we observed high coverage among the wealthiest and low coverage amongst the poorest family. For family planning health services, we observed slow improvement in the overall coverage and reduced inequity. We observed slow improvement in the coverage for treatment of childhood illness and saw high inequity persist among the poor and rich families.

 In Nepal, there are several ongoing efforts in the reproductive, maternal, new-born and child health policies and program (Boerma et al. 2018; KC et al. 2011; Ministry of Health 2004). Legislative framework exists to ensure free of cost immunization to all children residing in Nepal (Ministry of Law, Nepal 2016). Reproductive health rights are enshrined in the constitution of Nepal (Government of Nepal 2015). Continuous efforts to improve health programs involve strengthening the capacity by improving the capacity of the health workers, commodity management, health information systems and the strategy for health service delivery (Ministry of Health and Population 2016).

In the last 15 years, the national immunization program has made continuous successful efforts in improving access to health services through outreach and mobile clinics. Outreach and mobile clinics are additional clinics held oncertain days every month toprovide immunisation and childhood illness services in the community (Child Health Division MoHP 2016). As a result immunization coverage has improved in the lowest wealth quintiles and rural settings (KC et al. 2017). Through an established immunization supply chain system at central, sub-national, district and sub-district level, there was uninterrupted supply of vaccine to a larger extent in the last 15 years (Child Health Divsion MoHP 2017).

Health services are provided through outreach clinics in order to increase the coverage for the case management of childhood illness (Målqvist et al. 2017a). However, due to the functional status of the outreach clinics for management of childhood illness, the coverage has been slowly progressing. Also, since the health services provided by the private sector have been increasing, a tendency is seen among wealthier families to opt for private services while the poor relyon pharmacies.

In terms of maternal health services, the access to the health facilities increased primarily in urban areas since the beginning of 2000 and the access gradually increased in rural areas. Women received skilled birth attendence through the birthing centres located in these accessed health facilities (Målqvist et al. 2017b). Th indicators for the antenatal check-up and institutional delivery in Nepal improved after the introduction of the demand side financing for maternal health through the Safe Delivery Incentive Program (Ensor et al. 2017; Witter et al. 2011).

For family planning, a decade of slow progress in terms of intensification of modern method contraceptive use to prevent unwanted pregnancies is noted (Mehata et al. 2014; Shrestha et al. 2012). Nepal revitalised its commitment for family planning programming through the development and implementation of Nepal National Family Planning Costed Implementation Plan 2015–2020 (Ministry of Health and Population Nepal 2015). Prioritizing the resource allocation based on the decentralized planning and budgeting for RMNCH services will accelerate the improvement in the coverage and equity. Priorities need to be set based on the current coverage and equity for interventions.

There are some limitations in this study. First, the coverage estimate for intervention is based on the cross-sectional study design. However, DHS provides a nationally representative data and we acknowledge that any other design may not have been possible for the DHS surveys. Second, this survey only included married women, so the family planning coverage might have been underestimated. Third, the average annual rate of change is based on the coverage between 2001 and 2016, so the 5-yearly change between 2001–2006, 2006–2011 and 2011–2016 is masked. Lastly, LiST modelling does not include hospital-based interventions during the post neonatal period and the factors such as Gross National Income and female literacy which contribute to the reduction in mortality.

 The coverage and equity of maternal, new-born and child health intervention in Nepal has changed in pattern, based on the different strategies taken to strengthen the services. The successful implementation strategy for immunization program to improve the coverage can be used for reducing the inequity gap in the reproductive, maternal, neonatal and child health interventions. Context specific strategies such as to avert preventable maternal, neonatal, under five childhood deaths and stillbirths are required to accelerate the investment for improving coverage and equity for slowly progressing interventions.

## Electronic supplementary material

Below is the link to the electronic supplementary material.

Supplementary material 1 (DOCX 14 kb)

## Abbreviation

ANC: Antenatal care
ARC: Annualized rate of change
CCI: Composite Coverage Index
CPNM: Care seeking for pneumonia (CPNM)
DPT: Diphtheria pertussis and tetanus
FPS: Family planning service
LiST: Lives saving tools
NDHS: Nepal Demographic Health Survey
RMNCH: Reproductive, maternal, newborn and child health
SBA: Skilled birth attendance
SDG: Sustainable development goal
Td: Tetanus low dose diphtheria vaccination
