# Supplementary material for: Equity and Coverage in the Continuum of Reproductive, Maternal, Newborn and Child Health Services in Nepal-Projecting the Estimates on Death Averted Using the LiST Tool
Source: Matern Child Health J. 2019 Nov 30;24(Suppl 1):22–30. doi: 10.1007/s10995-019-02828-y (PMC7048704; doi:10.1007/s10995-019-02828-y)
Supplement: Supplementary file 1 — Supplementary material 1 (DOCX 14 kb) [file 10995_2019_2828_MOESM1_ESM.docx]

Coverage of RMNCH intervention by 2030 based on the ARC 2001-2016.

| Intervention | CPR of modern method | Unmet need | Family Planning need satisfied | ANC by skilled provider | Tetanus Toxoid 2+ doses | SBA assisted delivery | BCG vaccination | DPT-HiB-Hb vaccination | Measles Rubella vaccination | All basic vaccination | Vitamin A supplementation | Treatment of diarrhea by ORT and continued feeding | Care seeking for pneumonia | Access to improved water source |
| --- | --- | --- | --- | --- | --- | --- | --- | --- | --- | --- | --- | --- | --- | --- |
| 2016 | 43% | 24% | 64% | 86% | 65% | 63% | 98% | 86% | 90% | 78% | 83% | 61.40% | 84.90% | 95% |
| ARC | 1.3% | -1.1% | 0.9% | 7.7% | 2.4% | 11.7% | 1.0% | 1.2% | 1.7% | 1.1% |  | 2.5% | 8.2% | 0.7% |
| 2017 | 43.6% | 23.7% | 64.6% | 92.6% | 66.6% | 70.4% | 99.0% | 87.0% | 91.5% | 78.9% | 83.0% | 62.9% | 91.9% | 95.7% |
| 2018 | 44.1% | 23.5% | 65.2% | 99.8% | 68.2% | 78.6% | 99.0% | 88.1% | 93.1% | 79.7% | 83.0% | 64.5% | 91.9% | 96.3% |
| 2019 | 44.7% | 23.2% | 65.7% | 107.4% | 69.8% | 87.8% | 99.0% | 89.1% | 94.7% | 80.6% | 83.0% | 66.1% | 91.9% | 97.0% |
| 2020 | 45.3% | 23.0% | 66.3% | 115.7% | 71.5% | 98.1% | 99.0% | 90.2% | 96.3% | 81.5% | 83.0% | 67.8% | 91.9% | 97.7% |
| 2021 | 45.9% | 22.7% | 66.9% | 124.6% | 73.2% | 98.1% | 99.0% | 91.3% | 97.9% | 82.4% | 83.0% | 69.5% | 91.9% | 98.4% |
| 2022 | 46.5% | 22.5% | 67.5% | 134.2% | 74.9% | 98.1% | 99.0% | 92.4% | 97.9% | 83.3% | 83.0% | 71.2% | 91.9% | 98.4% |
| 2023 | 47.1% | 22.2% | 68.1% | 144.5% | 76.7% | 98.1% | 99.0% | 93.5% | 97.9% | 84.2% | 83.0% | 73.0% | 91.9% | 98.4% |
| 2024 | 47.7% | 22.0% | 68.8% | 155.7% | 78.6% | 98.1% | 99.0% | 94.6% | 97.9% | 85.1% | 83.0% | 74.8% | 91.9% | 98.4% |
| 2025 | 48.3% | 21.7% | 69.4% | 167.7% | 80.5% | 98.1% | 99.0% | 95.7% | 97.9% | 86.1% | 83.0% | 76.7% | 91.9% | 98.4% |
| 2026 | 48.9% | 21.5% | 70.0% | 180.6% | 82.4% | 98.1% | 99.0% | 96.9% | 97.9% | 87.0% | 83.0% | 78.6% | 91.9% | 98.4% |
| 2027 | 49.6% | 21.3% | 70.6% | 194.5% | 84.4% | 98.1% | 99.0% | 98.1% | 97.9% | 88.0% | 83.0% | 80.6% | 91.9% | 98.4% |
| 2028 | 50.2% | 21.0% | 71.3% | 209.5% | 86.4% | 98.1% | 99.0% | 98.1% | 97.9% | 88.9% | 83.0% | 82.6% | 91.9% | 98.4% |
| 2029 | 50.9% | 20.8% | 71.9% | 225.6% | 88.5% | 98.1% | 99.0% | 98.1% | 97.9% | 89.9% | 83.0% | 84.6% | 91.9% | 98.4% |
| 2030 | 51.5% | 20.6% | 72.6% | 243.0% | 90.6% | 98.1% | 99.0% | 98.1% | 97.9% | 90.9% | 83.0% | 86.8% | 91.9% | 98.4% |
